# Effectiveness of a Fully Automated Mobile Therapeutic Versus a General Chatbot in Reducing Depression and Anxiety and Improving Well-Being: Feasibility Randomized Controlled Trial

**DOI:** 10.2196/82642

**Published:** 2026-04-22

**Authors:** Barbora Kuta, Lukas Novak, Radka Zidkova, Jana Furstova, Klara Malinakova, Andrea De Winter, Vít Husek

**Affiliations:** 1 Palacký University Olomouc, Křížkovského 511/8, Olomouc, 779 00, Czech Republic, 420 773981876; 2 Faculty of Medical Sciences, University Medical Center Groningen, Groningen, The Netherlands

**Keywords:** depression, anxiety, well-being, conversational agents, digital intervention, randomized controlled trial, chatbot, solution-focused therapy

## Abstract

**Background:**

Given the increasing prevalence of depression and anxiety disorders and enduring barriers to care, there is a critical need for alternative treatment options. Generative artificial intelligence (AI) chatbots show promise for increasing access to mental health care, though more direct research is needed to establish their efficacy.

**Objective:**

This pilot study aimed to test the efficacy of a generative mental health chatbot rooted in solution-focused therapy compared to the general-purpose ChatGPT and an assessment-only control (AOC) group on depression, anxiety, and well-being.

**Methods:**

A total of 185 English-speaking adults were recruited online and randomly assigned to one of three groups: AI therapy, ChatGPT, or AOC. Of these, 147 eligible participants filled out a pretreatment assessment. Over a 3-week period, the AI therapy group (n=44) was instructed to complete 3 structured, fully automated app-based sessions per week (9 total), while the ChatGPT group (n=60) was instructed to engage in 9 unstructured conversations with ChatGPT (GPT-4o–based models). The control group (n=43) received no intervention. In the AI therapy group, 39% (n=17) completed all sessions, as did 62% (n=38) of those in the ChatGPT group. Primary outcome measures, self-assessed online at baseline and postintervention, included the Patient Health Questionnaire-9 (PHQ-9), Overall Depression Severity and Impairment Scale (ODSIS) (depression), 7-item Generalized Anxiety Disorder Scale (anxiety), and World Health Organization Well-Being Index (5-item version) (well-being). Linear mixed effects models were used for data analysis.

**Results:**

Compared to AOC, both the AI therapy group (*d*=−0.47; *P*=.01) and the ChatGPT group (*d*=−0.44; *P*=.02) demonstrated significant reductions in depression scores measured by PHQ-9. The AI therapy group showed nonsignificant reductions in anxiety (*d*=−0.37; *P*=.11) and ODSIS depression scores (*d*=−0.25; *P*=.22) and an increase in well-being (*d*=0.12; *P*=.53) compared to AOC. Similarly, a nonsignificant reduction in anxiety (*d*=−0.27; *P*=.22) and ODSIS depression scores (*d*=−0.12; *P*=.53) and an increase in well-being (*d*=0.20; *P*=.29) were observed in the ChatGPT group compared to AOC. The AI therapy group did not significantly outperform the ChatGPT group on any outcomes (PHQ-9: *b*=−0.19; *d*=0.03; *P*=.87; 7-item Generalized Anxiety Disorder Scale: *b*=−0.57; *d*=−0.11; *P*=.62; ODSIS: *b*=−0.59; *d*=−0.13; *P*=.50; and WHO: *b*=−0.38; *d*=−0.07; *P*=.69).

**Conclusions:**

Both the structured generative AI chatbot and ChatGPT showed a significant reduction in depression scores compared to the control group. No significant effects were observed across other outcomes, although descriptive trends indicated improvements in anxiety. While the AI therapy group showed descriptively better outcomes for depression and anxiety, differences between groups were not significant. A larger sample and longer intervention may be needed for the emerging trends to yield clinically meaningful effect sizes.

## Introduction

One in every 7 people met the criteria for a mental health disorder in 2021, with approximately 229 million people globally dealing with depression and 359 million with anxiety disorders [1]. Mental health illnesses have moved from the 9th to the 6th leading cause of disability-adjusted life years from 1990 to 2021 [2]. This shift highlights the growing societal burden of mental illness. Alongside the escalating prevalence of mental illness, the treatment gap is widening, with estimates indicating that fewer than 50% of adults with mental illness receive any form of mental health treatment, even in high-income countries [3].

It is estimated that 58% of people with clinical-level mental health issues do not seek any professional help [4], as access to care remains a salient barrier. Due to the lack of treatment services, only 23% of people affected by depression receive minimally adequate treatment according to current research standards in high-income countries, and even fewer (8%) receive such treatment in low and lower-middle-income countries [5]. Additionally, the waiting times for mental health services are prohibitive, averaging longer than 3 months [6,7]. Another barrier is financial affordability, though its impact has slightly decreased in recent years [8]. Moreover, in addition to these systemic factors, individual factors may also explain reticence to seek care. People affected by mental illness report a low perceived need for treatment, a desire to handle problems independently [9,10], or feeling too busy to pursue treatment [8]. In light of these findings, it is critical to establish treatment options that are affordable, easily accessible, and, if possible, short-term and effective.

Digital mental health interventions (DMHIs) tools are promising supplements and/or alternatives to traditional treatment that can enhance mental health care accessibility [11]. Digital modalities are gaining traction, including online psychotherapy and programs powered by virtual reality [12]. Anonymity is a key advantage of DMHIs; people who believe they are talking to a computer, relative to a human operator, are more willing to disclose or express sadness and have less fear of being evaluated [13]. Subsequently, DMHI tools have growing support for treating mental health challenges, including depression [14] and anxiety [15]. Moreover, when combined with traditional therapy, mental health apps that support patients between sessions can improve the effectiveness of usual treatment for both depressive and anxiety disorders [16].

With the rise of artificial intelligence (AI), particularly through the development of large language models, new possibilities have emerged to mediate the primary psychotherapeutic tool, conversation. Conversational agents (CAs), or chatbots, represent a promising, accessible, and affordable mental health tool that could automate some therapeutic procedures when the demand for professionals exceeds available capacity [17]. This leverages one of the primary benefits of DMHIs, which is the personalization of treatment, especially using machine learning [18], potentially leading to greater positive outcomes and lower dropout rates [11,19]. AI has reached a point where it is challenging to differentiate real conversations from those with CAs. When therapists were asked to distinguish between transcripts of interactions with human therapists and those with AI chatbots, they were correct only 53.9% of the time, performing no better than random guessing [20].

As early as 2019, at least 41 mental health chatbots were on the market, most of them claiming to provide therapy [21]. Yet, many of them had not been reviewed by professionals, placing them in a regulatory ‘gray area’ and raising questions of safety that have been discussed only by a few studies so far [22]. It is important to keep in mind that chatbots do carry potential risks; for example, they can “hallucinate,” have biases in judgment, or lack safety and quality control [23]. For professionals to accept and build trust in the new technology, it is crucial to have evidence-based tools [17,24], but rigorous research in this area has lagged behind AI’s rapid development. The advancement of generative artificial intelligence (GenAI) in the 2020s has significantly bolstered the capabilities of mental health CAs [25]. Although meta-analyses comparing randomized controlled trials (RCTs) on the effectiveness of CAs already exist, they include studies of different types of chatbots in terms of function (retrieval-based, rule-based, and generative) [26-29]. A systematic review and meta-analysis from 2024 showed a significant effect of CAs on depression but did not discuss the type of chatbot or AI model [26]. Another review and meta-analysis from 2023 showed similar positive results, but the majority of included studies examined retrieval-based CAs [27]. Even the most recent meta-analysis from 2025 on young people included only 3 studies examining GenAI CAs [29]. Therefore, as there are still only a few studies exploring the effect of GenAI chatbots on mental health, more evidence is needed to examine their effectiveness.

GenAI chatbots could help fill the treatment gap by supporting individuals who are waiting for therapy, who cannot afford therapy, and those at low or medium acuity levels who do not need intensive treatment or would not otherwise engage in therapy. The present study entails an early-stage pilot trial testing the merits of a GenAI therapy chatbot. As described below, the AI therapy chatbot tested in this pilot was trained to deliver solution-focused brief therapy (SFBT), a therapeutic approach selected for its strong fit with chatbot delivery. Specifically, SFBT offers a structured conversational format and emphasizes brief, goal-oriented interactions. In this pilot RCT, the AI therapy chatbot was compared to both an untrained ChatGPT-4o-based chatbot and an assessment-only control (AOC) group. In line with preregistered hypotheses, we plan to obtain preliminary estimates of potential changes, assuming that AI therapy will improve well-being, anxiety symptoms, depression symptoms, and functional impairment due to depressive symptoms compared to these 2 groups. Given the nonclinical and nontreatment-seeking sample, the study focused on short-term outcomes, including symptoms of depression and anxiety, as well as well-being indices.

## Methods

### Study Design and Participants

The preregistered study involved a pilot RCT in which participants were randomly assigned to 1 of three groups in a 1:1:1 ratio: (1) the AI therapy group, who engaged in AI-assisted therapy sessions via the ChatMind app with a requirement of 3 sessions per week for 3 weeks; (2) the ChatGPT group, who completed equivalent sessions with a general chatbot in the ChatGPT mobile app; and (3) the AOC group, who received no intervention. Participants were aware of their assigned groups so that they could perform the relevant tasks and were emailed twice a week to remind them about the experiment. Mental health indices were measured before and after the 3-week intervention protocols using an online self-assessment questionnaire hosted on the OQS platform.

The sample size was estimated based on a previous RCT [30] evaluating a 3-week solution-focused brief, human-delivered therapy intervention, as the AI therapy shares similar characteristics. Using the effect sizes from this study, a range of anticipated mean changes was estimated, and a simulation-based power analysis was conducted. Across different sample sizes, specifying a mixed-effects model accounting for repeated measures design and group differences, power simulations indicated that 16 participants per group would achieve ≥80% power to detect a very large effect size (*d*=1.24). Since we were preparing for a high dropout rate, which is common in mobile app research [31], we set the minimum number of participants in each group to 26. However, given the aims of this pilot-stage study, statistical power to detect a smaller effect was not the priority. More details on this analysis, including a power analysis report, are described in the project preregistration.

Participants were recruited using a convenience sampling method through online advertisements (eg, Instagram), emails, and push notifications in a partnered mental health app VOS. The VOS app shares the same parent company as ChatMind but is not an AI-based therapy chatbot. It targets a general, nonclinical population interested in mental well-being and provides general mental-well-being tools such as a mood tracker, guided journaling, breathing exercises, and meditations. Inclusion criteria were minimal; participants were eligible if they were native English-speaking, aged 18 years or older, were not being treated for any psychiatric condition, and had never used the ChatMind AI chatbot.

Participants were recruited in 2 waves. Enrollment for the first wave took place during November and December 2024 (prospective study registration took place on November 29, 2024); 83 participants were enrolled, but only 76 (92%) completed the baseline survey, of which 9 (12%) records were deleted for completing the questionnaire outside the time schedule or not finishing the questionnaire, resulting in 67 randomized participants in the first wave. To ensure a sufficient sample size for pilot-stage effect size estimates, an additional recruitment wave took place in February 2025. With the same randomization strategy, participants were allocated into groups. Of the 102 participants enrolled in this wave, 88 (86%) completed the baseline survey, of which 8 were incomplete or off-schedule records, resulting in 80 participants in the second wave and 147 participants in total. No safety incidents or adverse events were reported during the study. Consistent with the aims of a pilot RCT, statistical power was not prioritized; instead, the sample size was selected to enable estimation of preliminary effect sizes to provide early-stage evidence and foundational support for forthcoming large-scale trials.

The final analytic sample comprised 85 (58%) participants from the United States, 21 (14%) from Canada, and 41 (28%) from other predominantly English-speaking countries, including the United Kingdom, Ireland, and Australia. Participants ranged in age from 20 to 74 years (mean 38.4, SD 10.8), and 73% were women.

### Ethical Considerations

Participation was voluntary, with the only incentive being trial access to the ChatMind AI chatbot (ie, participants in all conditions got access after the 3-wk study period). Informed consent (Multimedia Appendix 1) was obtained from all individuals before the baseline assessment. To ensure privacy and confidentiality, all data were pseudonymized at the point of collection and stored on secure, password-protected servers. No personally identifiable information is reported in this study, and only aggregated data are presented to prevent the identification of individual participants. Individuals currently receiving psychiatric treatment were excluded to minimize the risk of distress or adverse reactions. The study design was approved by the Ethics Committee at Olomouc University Social Health Institute (OUSHI) (approval September 2, 2024).

### Measures

#### Anxiety Symptoms

Anxiety was measured using the 7-item Generalized Anxiety Disorder Scale (GAD-7), a 7-item scale assessing anxiety symptoms over the past 2 weeks, rated on a 4-point scale (0=*“Not at all”* to 3=*“Nearly every day”*). Total scores range from 0 to 21, with higher scores indicating greater anxiety severity [32]. Cronbach α was 0.89 using both baseline and postintervention data.

#### Depressive Symptoms

Depressive symptoms were evaluated using two scales: the Patient Health Questionnaire (PHQ-9) [33] and the Overall Depression Severity and Impairment Scale (ODSIS) [34]. The PHQ-9 assesses the severity of depressive symptoms over the past 2 weeks through 9 items based on the *Diagnostic and Statistical Manual of Mental Disorders, Fourth Edition* criteria for depression. Responses indicate the frequency of symptoms and are rated on a 4-point scale (0=*“Not at all”* to 3=*“Nearly every day”*). Total scores range from 0 to 27, with higher scores indicating more severe symptoms [33]. Cronbach α was 0.87 using both pre- and postintervention data. The ODSIS measures the severity and functional impairment of depression. Participants responded to items indicating symptom frequency on a 5-point scale (0=*“Not at all”* to 4=*“All the time”*). Total scores range from 0 to 20, whereby higher scores indicate greater impairment [34]. Based on both baseline and postintervention data, Cronbach α was 0.94.

#### Well-Being

The World Health Organization Well-Being Index (5-item version) (WHO-5) was used to assess subjective well-being. Participants indicated how often they had experienced any of the manifestations of well-being in the past 2 weeks on a 6-point scale (0=*“At no time”* to 5=*“All of the time”*), with total scores ranging from 0 to 25 and higher scores indicating greater well-being [35,36]. The Cronbach α of this tool was 0.90 using the baseline and postintervention data.

#### Attitude Toward AI

To obtain an indicative overview of participants’ perception of AI, we asked them which of the following attitudes they most related to: enthusiastic, open, neutral, skeptical, or negative.

For each scale, we used sum scores treated as continuous variables.

### Intervention

#### AI Therapy

Participants in the AI therapy group were given access to the ChatMind app, which provided AI therapy rooted in SFBT principles via voice and text messaging. A preview of the application is available in Multimedia Appendix 2. The app consists of 2 types of session lengths, roughly 10 and 30 minutes, although the final length depended on the flow of the conversation. Participants were instructed to complete 1 short session and 1 long session and a third session of their choice per week. Instructions for participants are available in Multimedia Appendix 3. The app incorporated an automated detection system for crisis-related language (eg, references to self-harm or suicidality) that redirected users to national helplines.

During each session, the AI therapy chatbot first identified the participant’s problem and expectations. Then it guided the participant to find a solution or a small step they could take to improve their situation. Conversations operated on GenAI, modified through prompt engineering and specialized architecture. Participants could choose to engage in oral or text communication. Participants were tracked using a unique promo code for using the app, which allowed us to determine whether users started at least 3 sessions per week (if more, it was considered valid, as starting a session did not mean that the respondent had completed it).

#### ChatGPT Group

Participants in this group were asked to download the ChatGPT mobile app, which allows both text and oral communication. From November 2024 to February 2025, free-tier ChatGPT users initially used the GPT-4o model, and after reaching their message limit, the system automatically switched them to the lighter GPT-4o-mini model. Therefore, for most conversations, the 4o model was used, and 4o-mini for the remainder. Participants were instructed to interact with ChatGPT 3 times a week for at least 10 minutes, as if they were talking to an AI therapy chatbot about whatever was bothering them. In terms of safety, we relied on ChatGPT’s in-built systems. To check that participants were completing their tasks, they had to provide confirmation each week.

#### Assessment-Only Control Group

Participants in the AOC group completed baseline and follow-up assessments 3 weeks later but did not receive any intervention components. AOC participants were instructed not to use any chatbot for psychological intervention during the 3-week study period.

### Statistical Analysis

Group differences in demographic variables and baseline differences in outcome measures were estimated using *χ*² tests for categorical variables and nonparametric analysis of variance (the Kruskal-Wallis test) for age. A *χ*² test was also performed to assess for differential attrition across the study groups. This test revealed a strong trend suggesting that dropout rates were dependent on group assignment (*χ*²=5.14; *P*=.08). This signal of differential attrition, driven by a substantially higher dropout rate in the AI therapy group, raises concerns about the validity of a per-protocol analysis (PPA), as this approach becomes susceptible to selection bias that can compromise the initial randomization. Thus, our primary analysis followed the intention-to-treat (ITT) principle to provide an unbiased estimate of the intervention’s effectiveness. A secondary PPA, consistent with one of the options outlined in our preregistration, was also conducted to explore the efficacy of the intervention specifically among participants who completed the study. The results of the PPA are presented in supplementary analysis in Multimedia Appendix 4.

To evaluate intervention effects, linear mixed-effects models were used. Separate models were fitted for each outcome variable: anxiety (GAD-7), depressive symptoms (PHQ-9), depression severity and impairment (ODSIS), and mental well-being (WHO-5). For the GAD-7, ODSIS, and WHO-5 outcomes, models were fitted with parameters estimated using restricted maximum likelihood. For the PHQ-9 outcome, initial diagnostic checks revealed significant heteroscedasticity. To address this, a mixed-effects model was refitted with the nonconstant variance explicitly modeled. All models were adjusted for participants’ education level to account for baseline differences across groups.

Each model included fixed effects for time, with measurements taken at baseline and after the 3-week study period (ie, preintervention vs postintervention), experimental group (AI therapy, ChatGPT, and AOC), and the time × group interaction representing the differential change over time by group. Random intercepts for participants accounted for individual differences in baseline scores and changes over time. Initially, AOC was coded as the reference group to derive contrasts between AOC and the 2 active treatment conditions, then we releveled the condition variable coding to directly contrast AI therapy and ChatGPT conditions via planned comparisons. Although the constructs measured together are often statistically related (eg, [37], they are conceptually different. Therefore, we treated the scales as independent variables (depression symptoms, anxiety symptoms, depression-related functional impairment, and positive well-being). Following strict adherence to our preregistration, we applied Holm-Bonferroni correction to the 8 preregistered directional hypotheses: 4 tests comparing AI therapy to control (across 4 outcomes) and 4 tests directly comparing AI therapy to ChatGPT (across 4 outcomes). For these 8 preregistered tests, 1-tailed *P*-values were computed based on the directional hypotheses (expecting AI therapy to show greater improvements). Comparisons between ChatGPT and control were not preregistered with directional hypotheses and are therefore reported as exploratory analyses with 2-tailed *P*-values and no correction for multiple testing.

To formally assess the mechanism of missing data, we performed an omnibus test for data being missing completely at random (MCAR). In the MCAR test, the assumptions of multivariate normality and homoscedasticity required for the standard parametric Little’s test were violated (Hawkins test, *P*<.001). Therefore, we relied on the robust nonparametric alternative. The result of this test was not statistically significant (*P*=.06), providing insufficient evidence from this omnibus test to reject the MCAR null hypothesis. To examine predictors of intervention completion (fidelity) and retention (completing the follow-up survey), we performed a series of logistic regression analyses. In these analyses, independent variables were outcome measures (ie, PHQ-9, ODSIS, WHO-5), age, gender, and attitude toward AI.

Effect sizes were reported as unstandardized regression coefficients (*b*) and Cohen *d*. Because all outcomes were self-reported by participants, blinding of outcome assessors was not feasible. The researchers conducting the data analysis were not blinded to group assignment; however, all code and analytic decisions were independently reviewed by multiple members of the research team to minimize potential bias. All statistical analyses were conducted using R software, version 4.3.0 (R Core Team, 2023) within the RStudio environment, version 2024.04.2.

## Results

### Participant Characteristics

**Figure 1.**
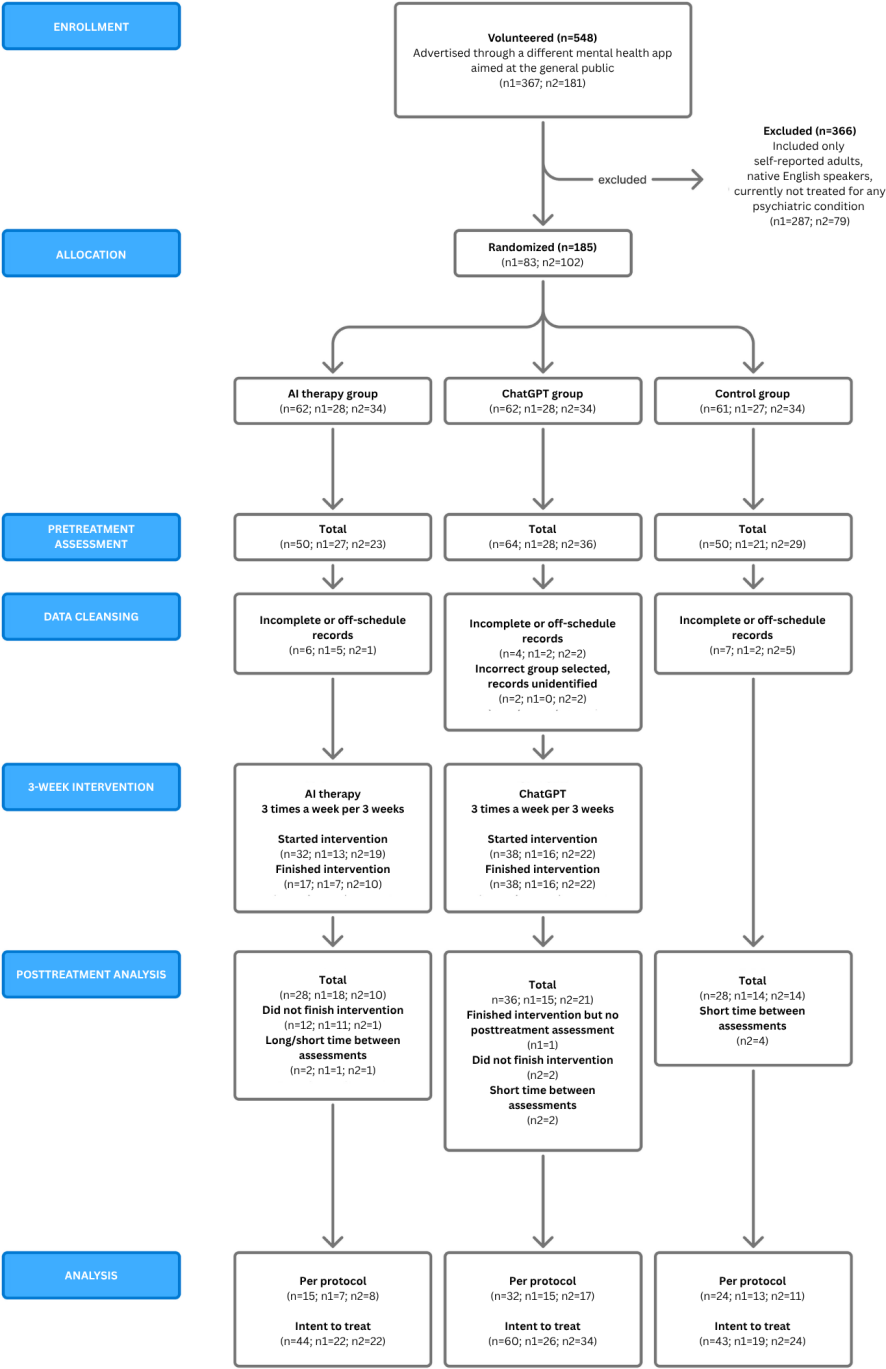
Consolidated Standards of Reporting Trials flow diagram. AI: artificial intelligence.

The Consolidated Standards of Reporting Trials (CONSORT) diagram chart in Figure 1 illustrates the process of respondent enrollment, allocation into groups, assessment, and intervention. Sample demographics and baseline characteristics for the ITT sample are provided in Table 1. Baseline comparisons showed no significant differences across the 3 groups for age or any of the clinical outcome measures (GAD-7, ODSIS, PHQ-9, or WHO-5), with all *P* values >.05. However, a Pearson χ² test with Monte Carlo simulated p-value revealed a significant difference in the distribution of education levels across the groups (χ² = 16.07, simulated p = .014). (*χ*²=16.07*; P*=.01). The demographic characteristics of the sample are summarized in Table 1.

**Table 1.** Sample characteristics by assigned group.

Variable	AI therapy (n=44)	ChatGPT (n=60)	Control (n=43)	Overall (n=147)
Age				
Mean (SD)	37 (9)	39 (10)	39 (13)	38 (11)
Median (IQR)	36 (23-71)	37 (20-74)	38 (22-73)	37 (20-74)
Gender, n (%)				
Female	28 (64)	42 (70)	37 (86)	107 (73)
Male	16 (36)	18 (30)	6 (14)	40 (27)
Country, n (%)				
United States	25 (57)	34 (57)	26 (60)	85 (58)
Canada	5 (11)	10 (17)	6 (14)	21 (14)
Other	14 (32)	16 (27)	11 (26)	41 (28)
Education, n (%)				
High school or less	6 (14)	5 (8.3)	13 (30)	24 (16)
Higher vocational	11 (25)	12 (20)	3 (7.0)	26 (18)
Bachelor’s degree	13 (30)	21 (35)	19 (44)	53 (36)
Master’s or PhD	14 (32)	22 (37)	8 (19)	44 (30)
Economic status, n (%)				
Not currently working	15 (34)	17 (28)	13 (30)	45 (31)
Employed	25 (57)	31 (52)	24 (56)	80 (54)
Self-employed	4 (9.1)	12 (20)	6 (14)	22 (15)
Recruitment phase, n (%)				
First	22 (50)	26 (43)	19 (44)	67 (46)
Second	22 (50)	34 (57)	24 (56)	80 (54)
Dropout participants, n (%)				
No	15 (34)	32 (53)	24 (56)	71 (48)
Yes	29 (66)	28 (47)	19 (44)	76 (52)

### Feasibility and Engagement

Full fidelity to the intervention protocol was relatively low in both treatment conditions; 17 (39%) of those in the AI therapy condition completed all intervention sessions, and 38 (62%) of those in the ChatGPT condition completed all sessions. Logistic regression revealed that none of the baseline clinical measures were significant predictors (GAD-7: odds ratio [OR]=0.97, *P*=.456; ODSIS: OR=0.95, *P*=0.23; PHQ-9: OR=0.98, *P*=.64). However, older age was significantly associated with a lower likelihood of completing the intervention (OR=0.95, *P*=.03).

Study retention, in terms of completing the follow-up survey, was 28 (64%) in the AI therapy condition, 36 (60%) in the ChatGPT condition, and 28 (65%) in the AOC condition. Logistic regression revealed that baseline symptom severity was not a significant predictor, although higher baseline ODSIS scores showed a trend toward predicting a lower likelihood of retention (OR=0.92; *P*=.05). In these models, older age again emerged as a significant predictor of lower retention (OR=0.95; *P*=.003). Furthermore, a more positive baseline attitude toward AI significantly predicted a higher likelihood of retention (OR=1.48; *P*=.048).

### Outcome Analysis

Descriptive statistics for anxiety, depression, and mental well-being scores at baseline and after the 3-week intervention period, stratified by the experimental groups, are presented in Multimedia Appendix 5. Kruskal-Wallis tests showed no significant group differences in mental health indices at baseline (*P*-value range: 0.43‐0.94). Although not a major feature of the trial, baseline assessment of attitudes toward AI-based therapy was very favorable (mean 3.05, SD 0.92) on a 4-point scale, where 1=skeptical or negative and 4=enthusiastic).

Table 2 presents the ITT intervention effect estimates, with unstandardized regression coefficients (*b*) for the interaction terms representing the estimated difference in change between groups from preintervention to postintervention. Table 3 presents the corresponding effect sizes (Cohen *d*) for these interaction effects, along with their 95% CIs.

**Table 2.** Results of mixed-effects models of temporal changes in anxiety (GAD-7^a^), depression (ODSIS^b^), (PHQ-9^c^), and mental well-being (WHO-5^d^) scores across study groups.^e^

	Anxiety (GAD-7)		Depression (ODSIS)		Depression (PHQ-9)		Mental well-being (WHO-5)	
Effect	*b*^f^ (95% CI)	*P* value	*b* (95% CI)	*P* value	*b* (95% CI)	*P* value	*b* (95% CI)	*P* value
Fixed effects								
Intercept	14.37 (10.53 to 18.21)	<.001	11.55 (7.99 to 15.11)	<.001	24 (19.69 to 28.30)	<.001	9.99 (5.75 to 14.23)	<.001
Time	0.38 (–1.32 to 2.08)	.66	0.35 (–0.93 to 1.64)	.58	0.64 (–0.60 to 1.89)	.31	–0.25 (–1.66 to 1.16)	.72
Experimental group (reference: control)								
AI^g^ therapy	–0.90 (–3.17 to 1.37)	.43	–0.22 (–2.27 to 1.84)	.83	–0.98 (–3.23 to 1.26)	.39	0.84 (–1.60 to 3.27)	.50
ChatGPT	0.23 (–1.89 to 2.36)	.83	0.13 (–1.79 to 2.06)	.89	0.28 (–2.01 to 2.56)	.81	–0.49 (–2.77 to 1.79)	.67
Interaction effects (reference: control group × time)								
AI therapy group × time	–1.98 (–4.39 to 0.43)	.05 (.37)	–1.13 (–2.94 to 0.68)	.11 (.66)	–2.67 (–4.74 to 0.60)	.006 (.046)	0.64 (–1.35 to 2.64)	.26 (>.99)
ChatGPT group × time	–1.41 (–3.68 to 0.85)	.22	–0.54 (–2.25 to 1.17)	.53	–2.47 (–4.61 to 0.34)	.02	1.02 (–0.86 to 2.90)	.28
Planned comparisons (reference: ChatGPT group × time)								
AI therapy group × time	–0.57 (–2.83 to 1.70)	.31 (>.99)	–0.59 (–2.29 to 1.12)	.25 (>.99)	–0.19 (–2.59 to 2.20)	.44 (>.99)	–0.38 (–2.26 to 1.50)	.66 (>.99)

a GAD-7: 7-item Generalized Anxiety Disorder Scale.

b ODSIS: Overall Depression Severity and Impairment Scale.

c PHQ-9: Patient Health Questionnaire-9.

d WHO-5: World Health Organization Well-Being Index (5-item version).

e Models also controlled for age, gender, country, education, employment status, and recruitment wave. *P* values for AI therapy group × time and AI therapy group × time (vs ChatGPT) are 1-tailed based on preregistered directional hypotheses; ChatGPT group × time comparisons are exploratory (not preregistered) and reported with 2-tailed *P* values. For preregistered tests, the main value represents the unadjusted 1-tailed *P* value, with the Holm-Bonferroni adjusted *P* value (corrected across 8 preregistered hypotheses) provided in bold parentheses. Exploratory tests show 2-tailed *P* values without adjustment.

f b: unstandardized regression coefficient.

g AI: artificial intelligence.

**Table 3.** Effect sizes (Cohen *d*, 95% CI) for intention-to-treat intervention effects.^a^

Effect	Anxiety (GAD-7^b^), Cohen *d*^f^ (95% CI)	Depression (ODSIS^c^), Cohen *d* (95% CI)	Depression (PHQ-9^d^), Cohen *d* (95% CI)	Mental well-being (WHO-5^e^), Cohen *d* (95% CI)
Interaction effects (reference: control group × time)				
AI^g^ therapy group × time	−0.37 (−0.83 to 0.08)	−0.25 (−0.66 to 0.15)	−0.47 (−0.84 to −0.11)	0.12 (−0.26 to 0.51)
ChatGPT group × time	−0.27 (−0.70 to 0.16)	−0.12 (−0.50 to 0.26)	−0.44 (−0.82 to −0.06)	0.20 (−0.17 to 0.56)
Planned comparisons (reference: ChatGPT group × time)				
AI therapy group × time	−0.11 (−0.54 to 0.32)	−0.13 (−0.51 to 0.25)	−0.03 (−0.46 to 0.39)	−0.07 (−0.44 to 0.29)

a Cohen *d* was calculated by dividing the unstandardized regression coefficient (*b*) and its CI by the pooled baseline SD. A negative *d* indicates a greater reduction in symptoms for the nonreference group.

b GAD-7: 7-item Generalized Anxiety Disorder Scale.

c ODSIS: Overall Depression Severity and Impairment Scale.

d PHQ-9: Patient Health Questionnaire-9.

e WHO-5: World Health Organization Well-Being Index (5-item version).

f b: unstandardized regression coefficient.

g AI: artificial intelligence.

During preregistered hypothesis testing, it was found that the AI therapy group exhibited a statistically significant reduction in PHQ-9 depressive symptoms compared to the control group with a 2.67 point greater reduction (*d*=−0.47, 1-tailed and noncorrected *P*=.006, corrected *P*=.046), which remained significant after Holm-Bonferroni correction for the 8 preregistered hypotheses. The AI therapy group also showed trends toward greater improvement in anxiety symptoms (GAD-7: 1.98 point reduction, *d*=−0.37) and ODSIS depression functional impairment scores (1.13 point reduction, *d*=−0.25), although these did not reach statistical significance. No significant change in well-being (as measured by the WHO-5) was observed compared to the control group.

During nonpreregistered exploratory analyses, we examined differences between the ChatGPT group and the control group in the outcome measures. It was revealed that the ChatGPT group showed a significant 2.47 point greater reduction in PHQ-9 depressive symptoms (*d*=-0.44, 2-tailed *P*=.02), as well as nonsignificant improvements in anxiety symptoms (*d*=-0.27) and ODSIS scores (*d*=-0.12). These exploratory results were not subject to multiple comparison correction. As shown in the supplemental analyses in Multimedia Appendix 4, these results were largely consistent with the per-protocol results.

The next set of models was the planned comparisons between the AI therapy and ChatGPT (reference group) conditions, as shown in the lower half of Tables2  3. Although none of the estimates reached statistical significance, the AI therapy group showed small, favorable effect size trends compared to the ChatGPT group for anxiety (*d*=-0.11), ODSIS depression severity and impairment scores (*d*=-0.13), PHQ depressive symptoms (*d*=-0.03), but not in well-being (*d*=-0.07). In the PPA, the ChatGPT group showed numerically larger reductions in depression scores (ODSIS and PHQ-9) compared to AI therapy, but these differences were very small and not statistically significant (Multimedia Appendix 4).

## Discussion

### Principal Findings

As digital therapy alternatives rapidly evolve, including GenAI-based therapy chatbots, there is a need for careful, iterative testing of these novel therapeutic modalities. This pilot study provides early-stage proof of concept for a brief 3-week (9 sessions) AI therapy chatbot intervention based on solution-focused principles (ie, ChatMind), relative to a standard untrained chatbot (GPT-4o-based models) condition and an AOC condition. As described below, findings provide foundational support for the overall promise/merits of GenAI chatbot therapy while also elucidating key areas of improvement regarding feasibility.

### Clinical Outcomes

#### Overview of Findings

Overall, both the AI therapy group and the ChatGPT group showed significant reductions in depressive symptoms and nonsignificant improvements in anxiety symptoms compared to the AOC. Changes in well-being scores were not significant in any group. Neither AI group significantly outperformed the other, although the AI therapy group exhibited greater descriptive changes. These findings should be interpreted as preliminary trends rather than definitive evidence of efficacy, given the small sample size and limited statistical power of the study.

#### Depression

Both the AI therapy and ChatGPT groups demonstrated a statistically significant reduction in depressive symptoms (PHQ-9), while functional impairment, as measured by ODSIS, remained unchanged. This suggests that AI interventions may influence symptom severity more than daily life functioning. Overall, the reduction in depressive symptoms aligns with previous meta-analyses highlighting the short-term effectiveness of CAs on depression [27]. The effect sizes achieved by the AI interventions on the PHQ-9 (AI therapy: *d*=−0.47; ChatGPT: *d*=−0.44) are comparable to those reported for psychotherapies for depression, which typically yield standardized mean differences ranging from 0.11 to 0.61 [38]. Although these reductions reached statistical significance, the mean changes in PHQ-9 scores (−2.7 points for AI therapy; −2.5 for ChatGPT) did not meet the commonly accepted minimal clinically important difference of approximately 3.3 points [39]. On both measures, the AI therapy group showed greater reductions, although these differences were not statistically significant compared to the ChatGPT group. The AI therapy intervention in this study, though based on GenAI, followed structured prompts emphasizing ventilation and goal setting inspired by SFBT, which has demonstrated effectiveness for depression [40]. This structured approach may have contributed to the greater descriptive improvements compared to the unstructured ChatGPT intervention.

#### Anxiety

Both the AI therapy group and the ChatGPT group demonstrated nonsignificant reductions in anxiety symptoms. The descriptive change fell below the minimal clinically important difference threshold of about 3.7 points [39]. A longer intervention may be required for these effects to reach statistical significance, although previous meta-analytic evidence suggests that CAs can have short-term effects on both generalized and specific anxiety [26,27]. By the nature of anxiety, the real-time availability of CAs may be 1 factor, making them effective in the short term. Furthermore, AI’s ability to detect and reframe cognitive distortions [41], which often contributes to anxiety, may help reduce its symptoms. Given AI’s current restriction to verbal communication, its potential may be strongest in text-based cognitive interventions. Importantly, this remains only a potential, and it is unclear whether AI can deliver such interventions without specific prompting. Recent research even suggests that GenAI models may themselves exhibit metaphorical “state anxiety” when exposed to trauma-related narratives [42], indicating that specific prompting or other adjustments may be necessary to optimize AI responses to anxious input.

#### Well-Being

In our study, both intervention groups showed slight, nonsignificant improvements in well-being, while the control group declined. The effect of CAs on well-being has been inconsistent across previous studies. Although a meta-analysis by Zhong et al shows short-term gains in well-being after a CA intervention [26], another of He et al does not [27]. Even though the WHO-5 questionnaire is sensitive to intervention-related changes [36], well-being is considered a relatively stable construct across the lifespan [43], which may limit the short-term impact of CAs. The therapeutic approach that is most commonly integrated into CAs, cognitive behavioral therapy [44], appears to be more effective in reducing negative affect than in enhancing positive affect [45]. This difference could explain the more consistent effects on depression and anxiety compared to well-being. In contrast, SFBT, which underpinned the AI therapy group, has demonstrated effectiveness for both affects [46]. Because cognitive behavioral therapy is often symptom-focused, its integration into CAs may be effective for reducing targeted issues like depression or anxiety, but less so for promoting overall well-being, highlighting the potential benefit of including elements from broader therapeutic frameworks.

#### Comparison of Two Chatbots

A key strength of our study is the direct comparison of a structured AI therapy chatbot with an unstructured general-purpose 1. Neither chatbot was found to significantly outperform the other in any outcome. In the ITT analysis, the AI therapy group showed slightly larger reductions than the ChatGPT group, with small effect sizes favoring AI therapy for anxiety (AI therapy: *d*=−0.37 vs ChatGPT: *d*=−0.27), ODSIS depression (AI therapy: *d*=−0.25 vs ChatGPT: *d*=−0.12), and PHQ depression (AI therapy: *d*=−0.47 vs ChatGPT: *d*=−0.44). However, in the per-protocol analysis, this pattern reversed for depression: the ChatGPT group achieved slightly greater reductions in PHQ-9 and ODSIS scores. This discrepancy may reflect differences in adherence or user engagement, as per-protocol analysis includes only participants who completed the intervention as intended. While the results of the ITT analysis may be closer to a real-life scenario, the per-protocol analysis is closer to the ideal situation. These differences should be interpreted with caution. However, they also highlight the need to examine which features, such as the AI model used, conversation flow, or therapeutic framing, drive effectiveness. We do not yet know what the determinants of the effectiveness of CAs are; however, recent studies suggest that prompt engineering influences their relevance, empathy, and contextual responses [33].

#### Factors Influencing Effectiveness

The emergence of GenAI represents a fundamental shift: unlike older retrieval-based or rule-based systems, generative models can produce original and coherent text [47]. Our findings revealed descriptive trends indicating that structured AI interactions led to greater results in depression and anxiety changes than in the ChatGPT group, raising questions about which features, such as the AI model used, conversation flow, or therapeutic framing, drive effectiveness. In line with our results, previous research suggests that prompt engineering influences the CA’s relevance, empathy, and contextual responses [33].

When we compared our results thoroughly with previous studies, we encountered several obstacles. While our study compared 2 generative chatbots with a control group, most earlier studies examined rule-based or retrieval-based CAs, limiting the generalizability of their findings to current models used in our trial. Moreover, many studies fail to report these crucial technical details, and even meta-analyses do not account for these differences [26,48]. Recent evidence indicates that GenAI chatbots may achieve larger effects on mental health outcomes compared to rule-based agents [28], and perform better in giving empathetic responses and establishing the alliance between the CA and the user [27]. Systematic comparisons between different types of CAs are therefore essential to identify which specific features drive the effectiveness of CAs.

### Feasibility

The multinational sample of nontreatment-seeking adults generally reported favorable attitudes toward AI-based therapy assessed at baseline; however, the suboptimal adherence to intervention protocols may suggest waning interest once involved. Indeed, only 39% of those in the AI therapy condition fully adhered to the 9-session protocol. This pattern may reflect limited engagement among participants without acute treatment needs and is an important consideration in our study of healthy, nontreatment-seeking samples. Fidelity was higher in the ChatGPT group (62%), likely due to several factors. Accessing ChatMind required extra steps, such as obtaining a promo code, which may have discouraged some participants. The structured, solution-focused session format may also have reduced engagement compared with the more flexible ChatGPT experience. Another possible contributing factor could have been differences in overall user-friendliness. Notably, participants with higher baseline depression severity and impairment on the ODSIS scale were more likely to adhere to the full protocol, although this trend did not reach statistical significance. However, this was not the case for higher depression symptom scores measured by the PHQ-9, suggesting greater adherence in those whose symptoms of depression had already begun to interfere with their lives. The relatively rigid structure of the current protocol (3 sessions per wk for 3 wk) may have also contributed to lower protocol adherence. In contrast, real-world applications of AI therapy are likely to be more flexible, allowing individuals to engage when most needed and at a preferred pace. Furthermore, this pilot tested a single therapeutic approach (SFBT), which may not resonate with all users or meet a wide range of needs and preferences. The fixed protocol may have felt repetitive for some participants, further limiting sustained engagement. Looking ahead, AI therapy tools will likely need to emulate the flexibility and adaptability of human therapists by tailoring interactions to patient preferences, symptom severity, and evolving goals to maximize engagement and therapeutic impact. Future feasibility research should focus on clinical, treatment-seeking samples, explore adaptive or flexible treatment models, and further evaluate the optimal number and pacing of AI therapy sessions.

### Strengths and Limitations

Our study is 1 of the few RCTs to investigate the effectiveness of a generative mental health chatbot. The field of AI is rapidly advancing, and our study focuses on instruments that use the most recent AI models, underscoring its urgency and relevance. To the best of our knowledge, we are the first to compare 2 generative chatbots against a control group, which is the strongest aspect of our study. Furthermore, the study code and data are freely available online so that findings from this study can be easily replicated.

The study also has several limitations. The first one is a high attrition rate, which is a common challenge in short-term CA intervention studies [49]. We addressed this limitation with second participant recruitment, but although the targeted sample size estimated by power analysis was achieved, a larger sample would still likely be needed to detect smaller effects. The second limitation is the intervention length. In addition, the second limitation relates to the nature of the GenAI on which both interventions were based, as it inherently limits control over the conversation flow and content compared to the earlier researched rule or retrieval-based chatbots. However, this also allowed for more naturalistic data and greater ecological validity. The third limitation is a lack of data on the exact length of interventions. We were not able to track the total time participants spent conversing within the AI therapy and ChatGPT groups. Nonetheless, this reflects a common limitation in studies of unsupervised digital interventions. The fourth limitation is that we did not specify in the guidelines that participants should not start any psychological treatment, only that they should not use another chatbot for therapeutic purposes. A final limitation of the present study is the absence of detailed engagement data, such as session duration or use of voice versus text input. Future studies should incorporate more precise tracking of user interaction patterns to better evaluate engagement and adherence.

### Implications

Future research should investigate the long-term effects of AI-based psychological interventions, as most studies, including ours, assess only short-term outcomes. Extended intervention periods and follow-up assessments are needed to evaluate sustainability and rule out novelty effects. Comparative studies should also explore different AI types, delivery formats (eg, text vs voice), and therapeutic approaches embedded in chatbots.

Regarding implications for practice, our results underscore that not all AI-based mental health tools are equal: therapeutic outcomes may critically depend on how AI is deployed in a particular chatbot, including the use of prompt engineering, conversation design, and alignment with established therapeutic frameworks. For chatbot developers, it will be important to build on development practices as well as psychological foundations that are supported by research, evaluate the effectiveness of specific chatbots through new research, and identify new factors contributing to their effectiveness.

### Conclusion

This study evaluated the effectiveness of a structured, generative chatbot rooted in solution-focused brief therapy compared to a general-purpose GenAI chatbot (ChatGPT) and a no-intervention control group over 3 weeks. Both the AI therapy and ChatGPT groups demonstrated a significant reduction in depressive symptoms compared to the control group. These findings support the potential of GenAI interventions for mental health. Further comparative studies are essential to identify the specific design features and therapeutic mechanisms that contribute to the effectiveness of AI-based mental health tools.

## Supplementary material

10.2196/82642 Multimedia Appendix 1 Informed consent.

10.2196/82642 Multimedia Appendix 2 ChatMind app screenshot.

10.2196/82642 Multimedia Appendix 3 Instructions.

10.2196/82642 Multimedia Appendix 4 Supplementary per-protocol analysis.

10.2196/82642 Multimedia Appendix 5 Descriptive characteristics.

10.2196/82642 Checklist 1 CONSORT-EHEALTH (V 1.6.1) checklist.

## Abbreviations

AI: artificial intelligence
AOC: assessment-only control
CA: conversational agent
CBT: cognitive behavioral therapy
CONSORT: Consolidated Standards of Reporting Trials
DMHI: digital mental health intervention
GAD-7: 7-item Generalized Anxiety Disorder Scale
GenAI: generative artificial intelligence
ITT: intention-to-treat
MCAR: missing completely at random
ODSIS: Overall Depression Severity and Impairment Scale
OR: odds ratio
PHQ-9: Patient Health Questionnaire-9
PPA: per-protocol analysis
RCT: randomized controlled trial
SFBT: solution-focused brief therapy
WHO-5: World Health Organization Well-Being Index (5-item version)
