# Supplementary material for: Effectiveness of a Fully Automated Mobile Therapeutic Versus a General Chatbot in Reducing Depression and Anxiety and Improving Well-Being: Feasibility Randomized Controlled Trial
Source: JMIR Ment Health. 2026 Apr 22;13:e82642. doi: 10.2196/82642 (PMC13102284; doi:10.2196/82642)
Supplement: Multimedia Appendix 1 [file mental-v13-e82642-s001.docx]

#### Informed consent and a detailed description of the research

#### Purpose of the research

As part of the scientific activities of the Institute of Social Health at Palacký University Olomouc (OUSHI) and in collaboration with the ChatMind application, we are currently conducting research focused on the effectiveness of AI-assisted therapy. We believe that a better understanding of this issue can help in the development of accessible psychological support.

#### How will the study look?

Participants in the study are divided into three groups: an experimental group undergoing a 3-week AI-assisted therapy program, a ChatGPT group interacting with ChatGPT, and a control group. All participants complete approximately 20-minute questionnaires before and after the experiment.

#### Why have we asked you to participate?

We have asked you because the data collected from you is very valuable for further research and recommendations.

#### What information will be collected?

We will collect information about:

1. basic demographic data
2. your attitudes and expectations regarding AI therapy
3. responses to psychological questionnaires

#### Will my participation in this study be confidential?

Yes, all information collected about you during this research will be confidential. You are not required to provide your name, and no individual will be identified at any time. Information in electronic form will be securely stored on computers or servers at UPOL and will be accessible only to the research team. No information will be shared with unauthorized individuals or third parties. If you wish, we can also provide you with the data you provide.

*Please note that, in the event of an investigation or court proceedings conducted by a legal authority, confidentiality may be overridden by legal orders. In such cases, the university will take appropriate legal measures to maintain confidentiality to the greatest extent possible.*

#### What will happen to the information you provide?

All information you provide will be stored at OUSHI in a manner that does not allow your identification. After the research is completed, the data will be stored on the servers of Palacký University Olomouc.

#### What will happen to the results?

The research will be summarized and presented as a summary of results and may be published in scientific journals. A copy of the research findings will be made available to you upon request.

#### What are the possible disadvantages of participation?

We do not anticipate any negative consequences for you as a result of participating.

#### What if there is a problem?

At the end of the questionnaire, we will provide you with contact details that you can use if you wish to discuss any issues related to completing the questionnaire.

#### Do you have any further questions?

For any further information, you can contact Barbora Kuta at barbora.kuta@oushi.upol.cz. If you agree to participate in this study, please answer the questions below and click the button to proceed to the questionnaire.

Thank you for taking the time to read this information.
