# Supplementary figures and images for: Effectiveness of a Fully Automated Mobile Therapeutic Versus a General Chatbot in Reducing Depression and Anxiety and Improving Well-Being: Feasibility Randomized Controlled Trial
Source: JMIR Ment Health. 2026 Apr 22;13:e82642. doi: 10.2196/82642 (PMC13102284; doi:10.2196/82642)

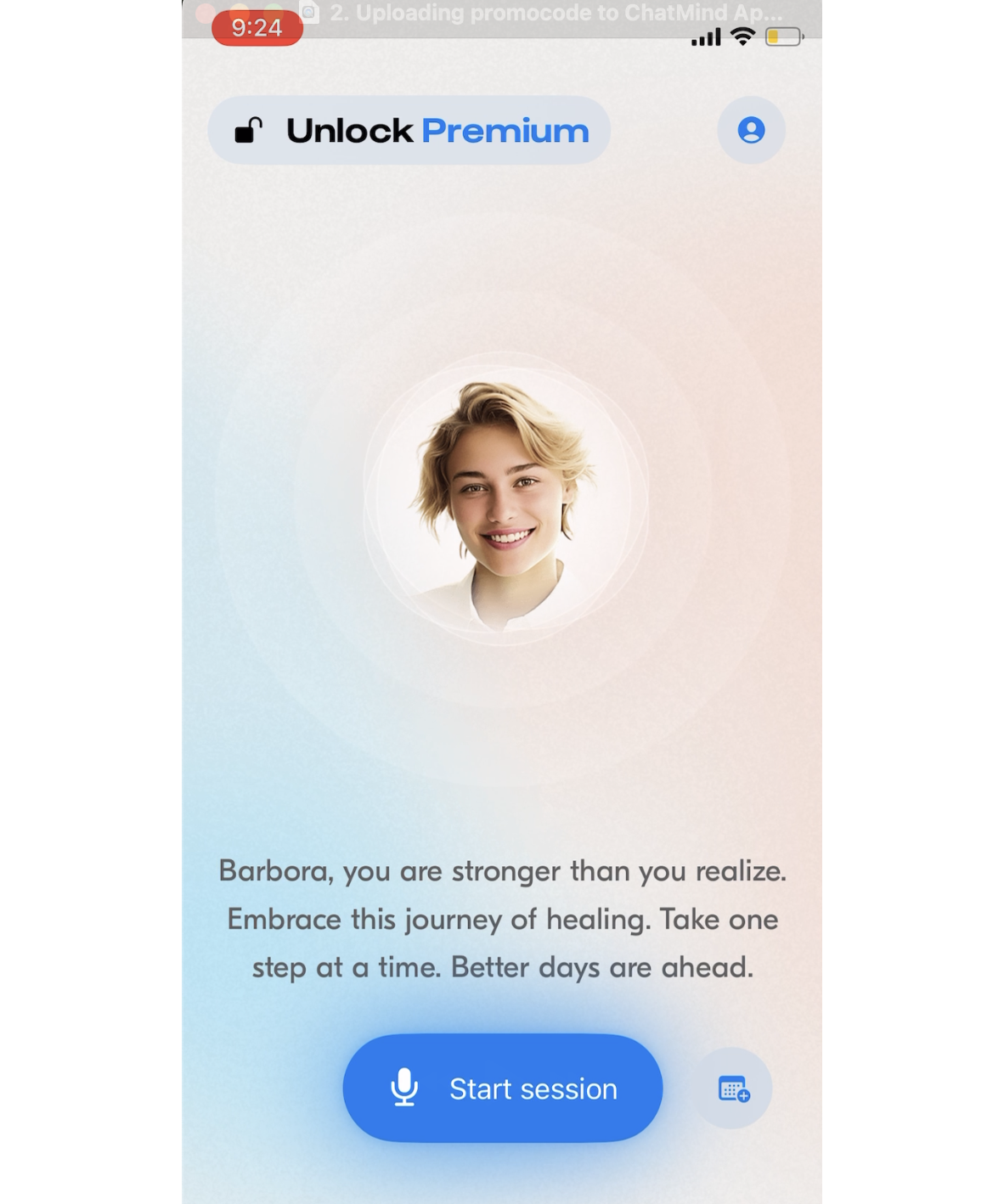

Supplement: Multimedia Appendix 2 [file mental-v13-e82642-s002.png]
