# Supplementary material for: Effectiveness of a Fully Automated Mobile Therapeutic Versus a General Chatbot in Reducing Depression and Anxiety and Improving Well-Being: Feasibility Randomized Controlled Trial
Source: JMIR Ment Health. 2026 Apr 22;13:e82642. doi: 10.2196/82642 (PMC13102284; doi:10.2196/82642)
Supplement: Multimedia Appendix 3 [file mental-v13-e82642-s003.docx]

## AI Therapy Group

**For the next three weeks:**

Use the ChatMind app three times a week.

Each week, complete at least one short session (10 minutes) and one long session (30 minutes). The third session can be your choice. You can choose which days to complete your sessions, but you may not do multiple sessions on the same day (e.g. regular sessions on Monday, short sessions on Wednesday and regular sessions on Saturday).

## ChatGPT

*The participants were instructed to use ChatGPT mobile app.*

**For the next three weeks:**

Use the ChatGPT app three times a week, treating it like an AI therapist. Discuss anything on your mind for at least 10 minutes per session. You can talk or text with the chatbot—whichever you prefer. Once a week you will be asked to confirm that you have completed 3 conversations with ChatGPT in a spreadsheet I will send you.

## Control

Please do not download or use the ChatMind app or any other similar AI therapy tools during the study, as this could affect the results.
