# Supplementary material for: Effectiveness of a Fully Automated Mobile Therapeutic Versus a General Chatbot in Reducing Depression and Anxiety and Improving Well-Being: Feasibility Randomized Controlled Trial
Source: JMIR Ment Health. 2026 Apr 22;13:e82642. doi: 10.2196/82642 (PMC13102284; doi:10.2196/82642)
Supplement: Multimedia Appendix 4 [file mental-v13-e82642-s004.zip › Multimedia Appendix 5. Supplementary Analysis.html]

AI Therapy -> Bara Kuta - PPA


# AI Therapy -> Bara Kuta - PPA

#### jf & Lukas Novak

#### 12 November, 2025

# 1. Setup and Installation

## 2. Data Load, Clean & Prepare for Analyses

# **Methods**

## **Participants and Procedure**

A total of 71 participants who completed the intervention protocol
were included in the primary per-protocol analysis. Participants were
randomly assigned to one of three groups: an AI-powered SFBT chatbot
(AI), a general-purpose chatbot (ChatGPT), or a waitlist control
group.

## **Statistical Analysis**

The primary analysis followed a **per-protocol**
approach, including only participants who completed the intervention.
Group differences in demographic variables and baseline differences in
outcome measures were estimated using chi-square tests for categorical
variables and non-parametric analysis of variance (the Kruskal-Wallis
test) for age. A chi-squared test was also performed to assess for
differential attrition across the study groups. This test revealed a
strong trend suggesting that dropout rates were dependent on group
assignment. This signal of differential attrition, driven by a
substantially higher dropout rate in the AI Therapy group, raises
concerns about the validity of a per-protocol analysis, as this approach
becomes susceptible to selection bias that can compromise the initial
randomization. Thus, our primary analysis followed the
intention-to-treat (ITT) principle to provide an unbiased estimate of
the intervention’s effectiveness. A secondary per-protocol analysis
(PPA), consistent with one of the options outlined in our
preregistration, was also conducted to explore the efficacy of the
intervention specifically among participants who completed the study.
The results of the PPA are presented in this document.

To evaluate intervention effects, linear mixed-effects models were
employed. Separate models were fit for each of the four primary outcome
variables: anxiety (GAD-7), depression (ODSIS, PHQ-9), and mental
well-being (WHO-5). For the ODSIS and WHO-5 outcomes, models were fit
using the `lme4` package, with parameters estimated using
restricted maximum likelihood (REML). For the GAD-7 and PHQ-9 outcomes,
initial diagnostic checks revealed significant heteroscedasticity. To
address this, mixed-effects models were fit using the
`glmmTMB` package, which models the non-constant variance
directly. All primary analyses were conducted on the original,
untransformed outcome scores to preserve direct clinical
interpretability.

Each model included fixed effects for time (pre-
vs. post-intervention), experimental group (AI therapy, ChatGPT, AOC),
recruitment wave, and the time✕group interaction, which represents the
differential change over time by group. Random intercepts for
participants accounted for individual differences. The control group was
initially coded as the reference group to derive contrasts between it
and the two active treatment conditions. Subsequently, the active
conditions were re-leveled to directly contrast the AI therapy and
ChatGPT groups via planned comparisons.

### **Multiple Comparison Correction**

Following strict adherence to our pre-registration, we applied
Holm-Bonferroni correction only to the 8 pre-registered directional
hypotheses: 4 tests comparing AI therapy to control (across 4 outcomes)
and 4 tests directly comparing AI therapy to ChatGPT (across 4
outcomes). For these 8 pre-registered tests, one-tailed p-values were
computed based on the directional hypotheses (expecting AI therapy to
show greater improvements). This procedure maintains strong control of
the family-wise error rate at α = 0.05 while offering greater
statistical power than a traditional Bonferroni correction.

Comparisons between ChatGPT and control were not pre-registered with
directional hypotheses and are therefore reported as **exploratory
analyses** with two-tailed p-values and no correction for
multiple testing. These exploratory results should be interpreted with
appropriate caution.

Sensitivity analyses restricted to the second recruitment wave
employed identical procedures to the primary analysis to ensure
consistency in error control across all analyses. Effect sizes were
reported as unstandardized regression coefficients (b) and Cohen’s
d. All statistical analyses were conducted using R software, version
4.3.0 (R Core Team, 2023) within the RStudio environment, version
2024.04.2.

# **Results**

## **Sample Characteristics**

Sample demographics for the per-protocol sample are provided in Table
1. Baseline comparisons showed no significant differences across the
three groups for age, any demographic variables, or any of the clinical
outcome measures (all p-values >.05), consistent with successful
randomization among those who completed the study.

**Table 1. Per-Protocol sample characteristics by assigned group**

| **Variable** | **Overall**  N = 711 | **Control**  N = 241 | **ChatGPT**  N = 321 | **AI Therapy**  N = 151 |
| --- | --- | --- | --- | --- |
| Age |  |  |  |  |
| Mean (SD) | 36 (11) | 38 (13) | 35 (8) | 37 (11) |
| Median [Min, Max] | 33 [20, 73] | 34 [22, 73] | 33 [20, 62] | 34 [25, 71] |
| Gender n (%) |  |  |  |  |
| Woman | 51 (72%) | 20 (83%) | 22 (69%) | 9 (60%) |
| Man | 20 (28%) | 4 (17%) | 10 (31%) | 6 (40%) |
| Country n (%) |  |  |  |  |
| USA | 34 (48%) | 12 (50%) | 16 (50%) | 6 (40%) |
| Canada | 12 (17%) | 4 (17%) | 6 (19%) | 2 (13%) |
| Other | 25 (35%) | 8 (33%) | 10 (31%) | 7 (47%) |
| Education n (%) |  |  |  |  |
| High school or less | 9 (13%) | 5 (21%) | 3 (9.4%) | 1 (6.7%) |
| Higher vocational | 13 (18%) | 1 (4.2%) | 9 (28%) | 3 (20%) |
| Bachelor degree | 26 (37%) | 12 (50%) | 10 (31%) | 4 (27%) |
| Master's or PhD | 23 (32%) | 6 (25%) | 10 (31%) | 7 (47%) |
| Recruitment phase n (%) |  |  |  |  |
| First | 35 (49%) | 13 (54%) | 15 (47%) | 7 (47%) |
| Second | 36 (51%) | 11 (46%) | 17 (53%) | 8 (53%) |
| Economic status n (%) |  |  |  |  |
| Not currently working2 | 16 (23%) | 6 (25%) | 7 (22%) | 3 (20%) |
| Employed | 44 (62%) | 15 (63%) | 18 (56%) | 11 (73%) |
| Self-Employed | 11 (15%) | 3 (13%) | 7 (22%) | 1 (6.7%) |
|  |  |  |  |  |
| --- | --- | --- | --- | --- |
| 1 n (%) | | | | |
| 2 Including students, unemployed, disabled pensioners, retirees, or those on maternity leave. | | | | |

## **Descriptive Outcome Statistics**

Descriptive statistics for the per-protocol sample are presented in
Table 2. The largest decrease in mean scores was observed in the AI
therapy group for anxiety symptoms (GAD-7), which dropped by 1.87
points, and for depressive symptoms as measured by the PHQ-9, with a
decrease of 1.53 points. The ChatGPT group also showed a notable
reduction of 2.09 points in PHQ-9 scores.

Table 2: Per-Protocol descriptive characteristics of clinical scores at baseline and post-intervention.

|  | Control | | ChatGPT | | AI Therapy | | Overall | |
| --- | --- | --- | --- | --- | --- | --- | --- | --- |
|  | Pretreatment (N=24) | Posttreatment (N=24) | Pretreatment (N=32) | Posttreatment (N=32) | Pretreatment (N=15) | Posttreatment (N=15) | Pretreatment (N=71) | Posttreatment (N=71) |
| Total anxiety score |  |  |  |  |  |  |  |  |
| Mean (SD) | 9.79 (4.74) | 10.0 (6.04) | 9.94 (5.42) | 8.50 (5.01) | 9.07 (6.13) | 7.20 (5.91) | 9.70 (5.30) | 8.75 (5.58) |
| Median [Min, Max] | 9.00 [2.00, 19.0] | 9.50 [1.00, 21.0] | 9.00 [1.00, 20.0] | 8.00 [1.00, 20.0] | 8.00 [2.00, 21.0] | 5.00 [0, 21.0] | 9.00 [1.00, 21.0] | 8.00 [0, 21.0] |
| Overall Depression Severity and Impairment Scale Score |  |  |  |  |  |  |  |  |
| Mean (SD) | 7.71 (4.39) | 8.42 (5.12) | 7.72 (4.89) | 7.56 (4.99) | 7.60 (3.56) | 7.73 (4.42) | 7.69 (4.41) | 7.89 (4.87) |
| Median [Min, Max] | 8.50 [0, 15.0] | 8.00 [0, 18.0] | 7.50 [0, 18.0] | 7.00 [0, 20.0] | 7.00 [2.00, 16.0] | 7.00 [0, 19.0] | 8.00 [0, 18.0] | 7.00 [0, 20.0] |
| Total depression score |  |  |  |  |  |  |  |  |
| Mean (SD) | 20.2 (5.65) | 21.3 (7.17) | 20.8 (6.53) | 18.7 (6.12) | 19.5 (5.50) | 18.0 (6.22) | 20.3 (5.97) | 19.4 (6.56) |
| Median [Min, Max] | 20.0 [10.0, 33.0] | 21.5 [9.00, 35.0] | 19.5 [11.0, 34.0] | 18.0 [10.0, 35.0] | 19.0 [13.0, 34.0] | 18.0 [10.0, 36.0] | 20.0 [10.0, 34.0] | 19.0 [9.00, 36.0] |
| Total well-being score |  |  |  |  |  |  |  |  |
| Mean (SD) | 9.29 (4.68) | 9.08 (5.85) | 9.47 (4.33) | 10.5 (4.84) | 12.0 (5.35) | 11.5 (5.55) | 9.94 (4.73) | 10.2 (5.35) |
| Median [Min, Max] | 10.0 [2.00, 18.0] | 9.00 [0, 19.0] | 10.5 [0, 17.0] | 11.0 [0, 19.0] | 11.0 [4.00, 20.0] | 12.0 [0, 17.0] | 10.0 [0, 20.0] | 11.0 [0, 19.0] |

## **Intervention Effects**

Table 3 presents the per-protocol intervention effect estimates, with
unstandardized regression coefficients (`b`) for the
interaction terms representing the estimated difference in change
between groups. Table 4 presents the corresponding effect sizes (Cohen’s
d) for these interaction effects, along with their 95% confidence
intervals.

Table 3: Per-Protocol results of mixed-effects models of temporal
changes in outcome scores.

|  | Anxiety (GAD-7) | | Depression (ODSIS) | | Depression (PHQ-9) | | Well-being (WHO-5) | |
| --- | --- | --- | --- | --- | --- | --- | --- | --- |
| Effect | b (95% CI)† | p‡ | b (95% CI)† | p‡ | b (95% CI)† | p‡ | b (95% CI)† | p‡ |
| **Fixed effects** | | | | | | | | |
| Intercept | 15.50 (10.65, 20.35) | <0.001 | 11.15 (5.76, 16.53) | <0.001 | 24.92 (18.99, 30.86) | <0.001 | 9.25 (3.49, 15.01) | 0.002 |
| Time | 0.25 (-1.14, 1.64) | 0.724 | 0.71 (-0.65, 2.07) | 0.303 | 1.08 (-0.23, 2.40) | 0.107 | -0.21 (-1.66, 1.24) | 0.776 |
| **Experimental Group** | | | | | | | | |
| Control | Reference |  | Reference |  | Reference |  | Reference |  |
| AI Therapy | -0.75 (-3.62, 2.12) | 0.608 | 0.46 (-2.90, 3.82) | 0.786 | 0.11 (-3.23, 3.46) | 0.947 | 1.81 (-1.78, 5.41) | 0.318 |
| ChatGPT | -0.35 (-2.97, 2.27) | 0.792 | -0.02 (-2.87, 2.83) | 0.988 | 0.51 (-2.65, 3.67) | 0.751 | -0.49 (-3.53, 2.56) | 0.751 |
| **Interaction effects** | | | | | | | | |
| Control Group × Time | Reference |  | Reference |  | Reference |  | Reference |  |
| AI Therapy Group × Time | -2.11 (-4.26, 0.05) | 0.028 (**0.194**) | -0.57 (-2.77, 1.63) | 0.303 (**1.000**) | -2.61 (-4.65, -0.57) | 0.006 (**0.049**) | -0.33 (-2.67, 2.02) | 0.609 (**1.000**) |
| ChatGPT Group × Time | -1.68 (-3.98, 0.63) | 0.154 | -0.86 (-2.66, 0.94) | 0.345 | -3.17 (-5.38, -0.96) | 0.005 | 1.27 (-0.65, 3.19) | 0.192 |
| **Planned Comparisons** | | | | | | | | |
| ChatGPT Group × Time (vs. AI) | Reference |  | Reference |  | Reference |  | Reference |  |
| AI Therapy Group × Time (vs. ChatGPT) | -0.43 (-2.90, 2.04) | 0.366 (**1.000**) | 0.29 (-1.80, 2.37) | 0.608 (**1.000**) | 0.56 (-1.81, 2.92) | 0.678 (**1.000**) | -1.60 (-3.82, 0.63) | 0.921 (**1.000**) |
|  |
| --- |
| Note:   Models also controlled for age, gender, country, education, employment status, and recruitment wave. †P-values for AI Therapy Group × Time and AI Therapy Group × Time (vs. ChatGPT) are one-tailed based on pre-registered directional hypotheses; ChatGPT Group × Time comparisons are exploratory (not pre-registered) and reported with two-tailed p-values. ‡For pre-registered tests, the main value represents the unadjusted one-tailed p-value, with the Holm-Bonferroni adjusted p-value (corrected across 8 pre-registered hypotheses) provided in bold parentheses. Exploratory tests show two-tailed p-values without adjustment. |
| a GAD-7 = Generalized Anxiety Disorder Scale b ODSIS = Overall Depression Severity and Impairment Scale c PHQ-9 = Patient Health Questionnaire d WHO-5 = Well-Being Index e b = Unstandardized Regression Coefficient f CI = Confidence Interval |

Table 4: Effect Sizes (Cohen’s d [95% CI]) for Per-Protocol Intervention
Effects.

|  | Anxiety (GAD-7) (a) | Depression (ODSIS) (b) | Depression (PHQ-9) (c) | Well-being (WHO-5) (d) |
| --- | --- | --- | --- | --- |
| Effect | d [95% CI] | d [95% CI] | d [95% CI] | d [95% CI] |
| **Interaction effects** | | | | |
| Control Group × Time | Reference | Reference | Reference | Reference |
| AI Therapy Group × Time | -0.40 [-0.81, 0.01] | -0.13 [-0.63, 0.37] | -0.44 [-0.78, -0.10] | -0.07 [-0.56, 0.43] |
| ChatGPT Group × Time | -0.32 [-0.75, 0.12] | -0.19 [-0.60, 0.21] | -0.53 [-0.90, -0.16] | 0.27 [-0.14, 0.67] |
| **Planned Comparisons** | | | | |
| ChatGPT Group × Time (vs. AI) | Reference | Reference | Reference | Reference |
| AI Therapy Group × Time (vs. ChatGPT) | -0.08 [-0.55, 0.39] | 0.07 [-0.41, 0.54] | 0.09 [-0.30, 0.49] | -0.34 [-0.81, 0.13] |
|  |
| --- |
| Note.  Cohen’s d calculated by dividing the unstandardized regression coefficient (b) and its confidence interval by the pooled baseline standard deviation. A negative d indicates a greater reduction in symptoms for the non-reference group. |
| a (a) GAD-7 = Generalized Anxiety Disorder Scale b (b) ODSIS = Overall Depression Severity and Impairment Scale c (c) PHQ-9 = Patient Health Questionnaire d (d) WHO-5 = Well-Being Index |

### **Primary Outcomes**

Following strict adherence to our pre-registration, we report the 8
pre-registered directional hypotheses with one-tailed p-values and
Holm-Bonferroni correction. The ChatGPT vs Control comparison is
reported as an exploratory finding.

**Pre-registered Hypotheses:** The AI Therapy group
demonstrated a statistically significant reduction in PHQ-9 depressive
symptoms compared to the control group (b = -2.61; d = -0.44, one-tailed
p = 0.006, adjusted p = 0.049), which remained significant after
Holm-Bonferroni correction for the 8 pre-registered hypotheses. The AI
Therapy group also showed trends toward greater reductions in anxiety
symptoms (GAD-7: b = -2.11; d = -0.4, one-tailed p = 0.028, adjusted p =
0.194), though this did not survive correction for multiple comparisons.
No significant effects were observed for ODSIS or WHO-5 outcomes (see
Table 3 for complete results). Direct comparisons between AI Therapy and
ChatGPT groups showed no statistically significant differences across
any outcome measure.

**Exploratory Finding (Not Pre-registered):** The
ChatGPT condition showed a statistically significant greater reduction
in PHQ-9 depressive symptoms relative to the control group (b = -3.17; d
= -0.53, two-tailed p = 0.005). This comparison was not pre-registered
with a directional hypothesis and should be interpreted with caution. It
is reported here for completeness but does not represent a confirmatory
test. —

# **Appendix A: Model Diagnostic Checks**

This appendix provides diagnostic checks for the primary per-protocol
linear models used in the main analysis.

### GAD-7 Model Diagnostics

#### Formal Test for Homoscedasticity

A temporary `lmer` model was fit to perform a
Breusch-Pagan test before proceeding with `glmmTMB`
diagnostics.

Table A 1 : Original homoscedasticity Test for GAD-7
Model.

| Breusch-Pagan Test p-value |
| --- |
| 0.03801181 |

This model was fit with `glmmTMB` to account for
heteroscedasticity. Diagnostics are based on simulated residuals from
the DHARMa package.

#### Linearity of Continuous Predictors

```
## `geom_smooth()` using formula = 'y ~ x'
```

#### Multicollinearity Assessment

Table A 2 : Variance Inflation Factors (VIFs) for the GAD-7
Model.


| Term | VIF | VIF\_CI\_low | VIF\_CI\_high | SE\_factor | Tolerance | Tolerance\_CI\_low | Tolerance\_CI\_high |
| --- | --- | --- | --- | --- | --- | --- | --- |
| Assigned\_Group | 1.80 | 1.51 | 2.24 | 1.16 | 0.56 | 0.45 | 0.66 |
| measurement\_phase | 2.37 | 1.94 | 2.99 | 1.54 | 0.42 | 0.33 | 0.51 |
| recruitment\_wave | 1.70 | 1.44 | 2.12 | 1.30 | 0.59 | 0.47 | 0.69 |
| Age | 1.26 | 1.12 | 1.58 | 1.12 | 0.79 | 0.63 | 0.90 |
| Gender | 1.19 | 1.07 | 1.51 | 1.09 | 0.84 | 0.66 | 0.94 |
| country\_4 | 1.88 | 1.57 | 2.34 | 1.17 | 0.53 | 0.43 | 0.64 |
| education\_5 | 1.80 | 1.51 | 2.24 | 1.10 | 0.56 | 0.45 | 0.66 |
| econ\_status\_3 | 1.38 | 1.20 | 1.71 | 1.08 | 0.73 | 0.58 | 0.83 |
| Assigned\_Group:measurement\_phase | 3.01 | 2.43 | 3.83 | 1.74 | 0.33 | 0.26 | 0.41 |

### ODSIS Model Diagnostics

#### Homoscedasticity and Normality of Residuals

Table A 3 : Homoscedasticity Test for ODSIS Model.

| Breusch-Pagan Test p-value |
| --- |
| 0.3736988 |

#### Linearity of Continuous Predictors

```
## `geom_smooth()` using formula = 'y ~ x'
```

#### Multicollinearity Assessment

Table A 4 : Variance Inflation Factors (VIFs) for the ODSIS
Model.


| Term | VIF | VIF\_CI\_low | VIF\_CI\_high | SE\_factor | Tolerance | Tolerance\_CI\_low | Tolerance\_CI\_high |
| --- | --- | --- | --- | --- | --- | --- | --- |
| Assigned\_Group | 1.67 | 1.40 | 2.10 | 1.29 | 0.60 | 0.48 | 0.71 |
| measurement\_phase | 2.96 | 2.37 | 3.81 | 1.72 | 0.34 | 0.26 | 0.42 |
| recruitment\_wave | 1.69 | 1.42 | 2.13 | 1.30 | 0.59 | 0.47 | 0.70 |
| Age | 1.27 | 1.12 | 1.61 | 1.13 | 0.79 | 0.62 | 0.89 |
| Gender | 1.13 | 1.03 | 1.52 | 1.06 | 0.88 | 0.66 | 0.97 |
| country\_4 | 1.82 | 1.52 | 2.30 | 1.35 | 0.55 | 0.43 | 0.66 |
| education\_5 | 1.65 | 1.39 | 2.08 | 1.28 | 0.61 | 0.48 | 0.72 |
| econ\_status\_3 | 1.35 | 1.18 | 1.71 | 1.16 | 0.74 | 0.59 | 0.85 |
| Assigned\_Group:measurement\_phase | 3.52 | 2.78 | 4.55 | 1.87 | 0.28 | 0.22 | 0.36 |

### PHQ-9 Model Diagnostics

#### Formal Test for Homoscedasticity

A temporary `lmer` model was fit to perform a
Breusch-Pagan test before proceeding with `glmmTMB`
diagnostics.

Table A 5 : Original homoscedasticity Test for PHQ-9
Model.

| Breusch-Pagan Test p-value |
| --- |
| 0.009312744 |

This model was fit with `glmmTMB` to account for
heteroscedasticity. Diagnostics are based on simulated residuals from
the DHARMa package.

#### Linearity of Continuous Predictors

```
## `geom_smooth()` using formula = 'y ~ x'
```

#### Multicollinearity Assessment

Table A 6 : Variance Inflation Factors (VIFs) for the PHQ-9
Model.


| Term | VIF | VIF\_CI\_low | VIF\_CI\_high | SE\_factor | Tolerance | Tolerance\_CI\_low | Tolerance\_CI\_high |
| --- | --- | --- | --- | --- | --- | --- | --- |
| Assigned\_Group | 1.55 | 1.33 | 1.93 | 1.12 | 0.65 | 0.52 | 0.75 |
| measurement\_phase | 2.35 | 1.93 | 2.97 | 1.53 | 0.43 | 0.34 | 0.52 |
| recruitment\_wave | 1.69 | 1.43 | 2.11 | 1.30 | 0.59 | 0.47 | 0.70 |
| Age | 1.28 | 1.13 | 1.60 | 1.13 | 0.78 | 0.63 | 0.88 |
| Gender | 1.14 | 1.04 | 1.49 | 1.07 | 0.88 | 0.67 | 0.96 |
| country\_4 | 1.85 | 1.55 | 2.31 | 1.17 | 0.54 | 0.43 | 0.65 |
| education\_5 | 1.71 | 1.45 | 2.13 | 1.09 | 0.58 | 0.47 | 0.69 |
| econ\_status\_3 | 1.41 | 1.23 | 1.75 | 1.09 | 0.71 | 0.57 | 0.82 |
| Assigned\_Group:measurement\_phase | 2.64 | 2.15 | 3.34 | 1.62 | 0.38 | 0.30 | 0.47 |

### WHO-5 Model Diagnostics

#### Homoscedasticity and Normality of Residuals

Table A 7 : Homoscedasticity Test for WHO-5 Model.

| Breusch-Pagan Test p-value |
| --- |
| 0.8297952 |

#### Linearity of Continuous Predictors

```
## `geom_smooth()` using formula = 'y ~ x'
```

#### Multicollinearity Assessment

Table A 8 : Variance Inflation Factors (VIFs) for the WHO-5
Model.


| Term | VIF | VIF\_CI\_low | VIF\_CI\_high | SE\_factor | Tolerance | Tolerance\_CI\_low | Tolerance\_CI\_high |
| --- | --- | --- | --- | --- | --- | --- | --- |
| Assigned\_Group | 1.66 | 1.40 | 2.10 | 1.29 | 0.60 | 0.48 | 0.71 |
| measurement\_phase | 2.96 | 2.37 | 3.81 | 1.72 | 0.34 | 0.26 | 0.42 |
| recruitment\_wave | 1.69 | 1.42 | 2.13 | 1.30 | 0.59 | 0.47 | 0.70 |
| Age | 1.27 | 1.12 | 1.61 | 1.13 | 0.79 | 0.62 | 0.89 |
| Gender | 1.13 | 1.03 | 1.52 | 1.06 | 0.88 | 0.66 | 0.97 |
| country\_4 | 1.82 | 1.52 | 2.30 | 1.35 | 0.55 | 0.43 | 0.66 |
| education\_5 | 1.65 | 1.39 | 2.08 | 1.28 | 0.61 | 0.48 | 0.72 |
| econ\_status\_3 | 1.35 | 1.18 | 1.71 | 1.16 | 0.74 | 0.59 | 0.85 |
| Assigned\_Group:measurement\_phase | 3.51 | 2.78 | 4.54 | 1.87 | 0.28 | 0.22 | 0.36 |

# **Appendix c: Sensitivity Analysis on Recruitment Wave**

To address concerns about potential differences between the two
recruitment waves, we conducted a sensitivity analysis by re-running the
primary mixed-effects models using only data from the second wave of
participants (n = 36). This analysis aimed to determine if the
intervention effects would be stable and not be unduly influenced by
potential issues with the initial recruitment cohort.

The results of this sensitivity analysis, presented in Table 5, were
largely consistent with the primary per-protocol analysis. While most of
the effects were no longer statistically significant after
Holm-Bonferroni correction, which is expected given the reduced sample
size and lower statistical power, the overall pattern of results
remained the same. However, the reduction in PHQ-9 depressive symptoms
in the AI Therapy group remained statistically significant even after
correction (b = -4.61, adjusted p = 0.003). As an exploratory finding,
the ChatGPT group also showed a reduction (b = -4.56, two-tailed p =
0.013), though this was not pre-registered and should be interpreted
with caution. For other outcomes, the patterns of effects were also
similar to the main analysis, with no new significant differences
emerging. This consistency suggests that the study’s main conclusions
are robust. The full results are detailed in Table 5.

Table 5: Sensitivity Analysis Results of Mixed-Effects Models (Second
Recruitment Wave Only).

|  | Anxiety (GAD-7) | | Depression (ODSIS) | | Depression (PHQ-9) | | Well-being (WHO-5) | |
| --- | --- | --- | --- | --- | --- | --- | --- | --- |
| Effect | b (95% CI)† | p‡ | b (95% CI)† | p‡ | b (95% CI)† | p‡ | b (95% CI)† | p‡ |
| **Fixed effects** | | | | | | | | |
| Intercept | 20.21 (16.32, 24.11) | <0.001 | 12.60 (5.76, 19.45) | <0.001 | 24.38 (17.41, 31.34) | <0.001 | 9.22 (2.11, 16.32) | 0.013 |
| Time | -0.00 (-1.45, 1.45) | 1.000 | 1.18 (-1.06, 3.43) | 0.292 | 2.00 (0.22, 3.78) | 0.028 | -1.64 (-3.88, 0.61) | 0.148 |
| **Experimental Group** | | | | | | | | |
| Control | Reference |  | Reference |  | Reference |  | Reference |  |
| AI Therapy | -4.90 (-7.71, -2.08) | <0.001 | -2.69 (-7.09, 1.70) | 0.221 | -2.74 (-6.26, 0.79) | 0.128 | 3.87 (-0.68, 8.42) | 0.093 |
| ChatGPT | -2.17 (-5.40, 1.07) | 0.190 | -1.62 (-5.73, 2.49) | 0.428 | -1.16 (-5.34, 3.02) | 0.586 | -0.99 (-5.25, 3.27) | 0.639 |
| **Interaction effects** | | | | | | | | |
| Control Group × Time | Reference |  | Reference |  | Reference |  | Reference |  |
| AI Therapy Group × Time | -2.85 (-5.71, 0.02) | 0.026 (**0.181**) | -1.80 (-5.26, 1.66) | 0.149 (**0.896**) | -4.61 (-7.31, -1.90) | <0.001 (**0.003**) | 0.01 (-3.45, 3.47) | 0.499 (**1.000**) |
| ChatGPT Group × Time | -2.25 (-5.75, 1.25) | 0.207 | -1.82 (-4.70, 1.07) | 0.209 | -4.56 (-8.18, -0.95) | 0.013 | 2.45 (-0.43, 5.34) | 0.093 |
| **Planned Comparisons** | | | | | | | | |
| ChatGPT Group × Time (vs. AI) | Reference |  | Reference |  | Reference |  | Reference |  |
| AI Therapy Group × Time (vs. ChatGPT) | -0.59 (-4.63, 3.44) | 0.387 (**1.000**) | 0.02 (-3.18, 3.21) | 0.505 (**1.000**) | -0.04 (-3.79, 3.71) | 0.491 (**1.000**) | -2.45 (-5.64, 0.75) | 0.936 (**1.000**) |
|  |
| --- |
| Note:   Models controlled for age, gender, country, education, and employment status. †P-values for AI Therapy Group × Time and AI Therapy Group × Time (vs. ChatGPT) are one-tailed based on pre-registered directional hypotheses; ChatGPT Group × Time comparisons are exploratory (not pre-registered) and reported with two-tailed p-values. ‡For pre-registered tests, the main value represents the unadjusted one-tailed p-value, with the Holm-Bonferroni adjusted p-value (corrected across 8 pre-registered hypotheses) provided in bold parentheses. Exploratory tests show two-tailed p-values without adjustment. |
