# Supplementary material for: Effectiveness of a Fully Automated Mobile Therapeutic Versus a General Chatbot in Reducing Depression and Anxiety and Improving Well-Being: Feasibility Randomized Controlled Trial
Source: JMIR Ment Health. 2026 Apr 22;13:e82642. doi: 10.2196/82642 (PMC13102284; doi:10.2196/82642)
Supplement: Multimedia Appendix 5 [file mental-v13-e82642-s005.docx]

Descriptive characteristics of the scores of anxiety, depression, and mental well-being at baseline and after the three-week intervention period (i.e., pre- and post-intervention), stratified by intervention group.

|  | AI therapy group | |  | ChatGPT group | |  | Control group | |
| --- | --- | --- | --- | --- | --- | --- | --- | --- |
| Variable | Pre | Post |  | Pre | Post |  | Pre | Post |
| No. of observations (N) | 44 | 28 |  | 60 | 36 |  | 43 | 28 |
| Anxiety (GAD-7)^a^ |  |  |  |  |  |  |  |  |
| Mean (SD) | 9.09 (5.35) | 8.07 (5.88) |  | 9.65 (5.50) | 8.83 (5.18) |  | 10.1 (4.96) | 9.96 (5.67) |
| Median [Min, Max] | 7.50 [2.00, 21.0] | 7.00 [0, 21.0] |  | 9.00 [1.00, 21.0] | 8.00 [1.00, 20.0] |  | 9.00 [0, 21.0] | 9.50 [1.00, 21.0] |
| Depression (ODSIS)^b^ |  |  |  |  |  |  |  |  |
| Mean (SD) | 8.16 (4.25) | 7.46 (4.69) |  | 8.17 (4.78) | 7.47 (5.01) |  | 8.51 (4.37) | 8.25 (4.84) |
| Median [Min, Max] | 8.00 [0, 19.0] | 7.50 [0, 19.0] |  | 8.00 [0, 18.0] | 7.00 [0, 20.0] |  | 8.00 [0, 20.0] | 8.00 [0, 18.0] |
| Depression (PHQ-9)^c^ |  |  |  |  |  |  |  |  |
| Mean (SD) | 19.9 (5.00) | 18.5 (6.06) |  | 20.6 (6.11) | 18.8 (6.10) |  | 21.2 (5.59) | 21.0 (6.75) |
| Median [Min, Max] | 19.5 [11.0, 34.0] | 18.5 [9.00, 36.0] |  | 20.0 [10.0, 34.0] | 18.0 [10.0, 35.0] |  | 21.0 [10.0, 36.0] | 21.5 [9.00, 35.0] |
| Mental Well-being (WHO-5)^d^ |  |  |  |  |  |  |  |  |
| Mean (SD) | 11.0 (5.55) | 11.6 (6.67) |  | 9.58 (4.71) | 10.6 (5.04) |  | 9.56 (5.39) | 9.36 (5.58) |
| Median [Min, Max] | 10.0 [1.00, 23.0] | 12.0 [0, 25.0] |  | 9.00 [0, 22.0] | 11.0 [0, 22.0] |  | 10.0 [1.00, 25.0] | 10.0 [0, 19.0] |

^a^GAD-7 = Generalized Anxiety Disorder Scale

^b^ODSIS = Overall Depression Severity and Impairment Scale

^c^PHQ-9 = Patient Health Questionnaire

^d^WHO-5 = Well-Being Index
